# Antidepressant Potential of Cinnamic Acids: Mechanisms of Action and Perspectives in Drug Development

**DOI:** 10.3390/molecules24244469

**Published:** 2019-12-06

**Authors:** Lúcio Ricardo Leite Diniz, Marilia Trindade de Santana Souza, Joice Nascimento Barboza, Reinaldo Nóbrega de Almeida, Damião Pergentino de Sousa

**Affiliations:** 1 Departament of Pharmacy, Federal University of Sergipe, São Cristóvão SE 49100-000, Brazil; luciodiniz@yahoo.com.br (L.R.L.D.); biomari@hotmail.com (M.T.d.S.S.); 2 Departament of Pharmaceutical Sciences, Federal University of Paraíba, João Pessoa PB 58051-970, Brazil; joicenascimentobarboza@gmail.com; 3 Department of Physiology and Pathology, Federal University of Paraíba, João Pessoa PB 58051-970, Brazil; reinaldoan@uol.com.br

**Keywords:** natural products, phenylpropanoids, phenolic acids, plants, depression, behavioral disorders, forced swim test, tail suspension test

## Abstract

Depression is a health problem that compromises the quality of life of the world′s population. It has different levels of severity and a symptomatic profile that affects social life and performance in work activities, as well as a high number of deaths in certain age groups. In the search for new therapeutic options for the treatment of this behavioral disorder, the present review describes studies on antidepressant activity of cinnamic acids, which are natural products found in medicinal plants and foods. The description of the animal models used and the mechanisms of action of these compounds are discussed.

## 1. Introduction

Depression is a widespread chronic psychiatric disease, characterized by low mood, lack of energy, sadness, insomnia, and high morbidity, that affects more than 300 million people worldwide [1,2]. This illness can affect anyone, regardless of age, sex, social status, education, nationality or ethnic origin [3,4]. It is the leading cause of disability and is directly associated with a remarkable number of suicides cases around the world [5,6].

Despite the physiopathological mechanism of depression remaining not widely elucidated and unclear, numerous studies have shown a multifactorial origin, involving genetics, environmental, psychological, and social factors, as well as dysfunction in multiple brain areas such as the hippocampus, prefrontal cortex, nucleus accumbens, and amygdale [7,8,9,10]. Recent findings of the presence of inflammatory process and oxidative stress in the pathophysiology of depression provide new pathways and treatment targets for improvement of pharmacological approach in depression [11]. Recently, Réus et al. (2019) have reported a microglial activation with formation of intracellular multiprotein complexes, the inflammasomes, which in turn activate interleukin-1β (IL-1β) that leads to a significant increase in the production and expression of tumor necrosis factor-α (TNF-α), IL-1β, reactive species of oxygen (ROS), and nitric oxide (NO) [12]. This finding is in accordance with a previous study performed by Oglodek (2017), in which it was identified that major depressive disorders, associated or not to posttraumatic stress disorder, present changes in the cytokines and increased oxidative stress [13].

**Figure 1:**
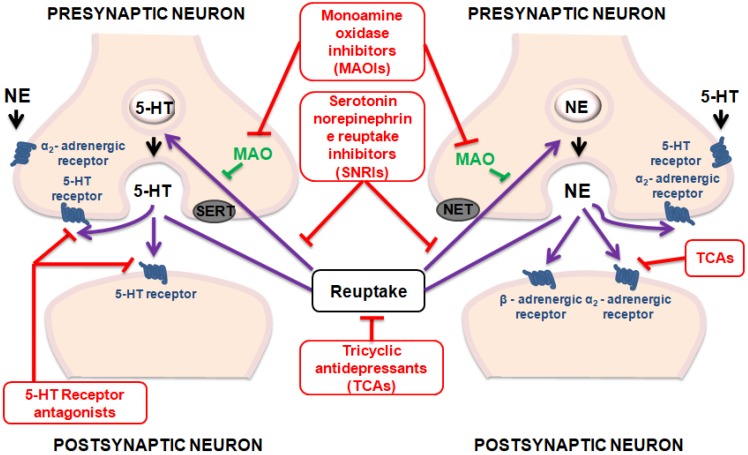
Mechanisms of action of antidepressant drugs.

Although efforts to increase knowledge and skills for healthcare providers have been made, depression remains both underdiagnosed and undertreated [14]. Actually, the therapeutics tools used in the treatment of depression have not produced satisfactory outcomes as necessary novel strategies to improve treatment outcomes [15,16]. Figure 1 summarizes the main mechanisms of action of antidepressant drugs.

**Figure 2:**
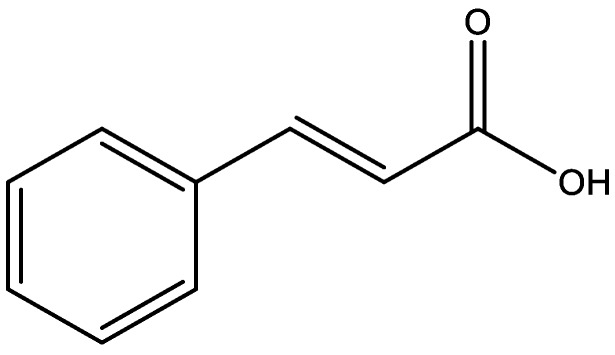
Chemical structure of cinnamic acid.

Natural products have been important sources of new drugs against various pathologies. Reports of antidepressant activity on these compounds indicate that they may be an alternative treatment option for depression [17]. Cinnamic acids are a group of aromatic carboxylic acids with carbonic skeleton C_6_–C_3_ (Figure 2) found in a variety of plants and foods, for which the biosynthetic route can generate several secondary metabolites such as coumarins, lignans, isoflavonoids, flavonoids, and others natural products [18].Cinnamic acids and their derivatives have attracted the attention of researchers due to their wide distribution in nature, low toxicity, structural diversity, and pharmacological actions [19], as anti-inflammatory [20], antioxidant [21], antitumor [22], hypoglycemic [23], antidepressant [24], and cytoprotective actions of neuroinflammation in neurodegenerative diseases [25]. Considering the importance of cinnamic acids as bioactive substances and their presence in various foods and medicinal plants, this review discusses the antidepressant action mechanisms of these compounds, demonstrating their therapeutic potential for depressive disorders.

## 2. Materials and Methods

The present study was carried out based on the literature review of cinnamic acids with antidepressant activity. The survey, conducted in the Pubmed database, for studies published from January 2002 to October 2019, used the following keywords: Cinnamic acid, coumaric acid, para-coumaric acid, meta-coumaric acid, ortho-coumaric acid, ferulic acid, caffeic acid, sinapic acid, trimethoxycinnamic acid, methylenedioxycinnamic acid, methoxycinnamic acid, dimethoxycinnamic acid, antidepressant, and depression. The scientific publications were selected from studies published in English language.

## 3. Antidepressant Activity of Cinnamic Acids

Based on increasing evidence of the contributions of neuronal pro-inflammatory mediators and oxidative stress in the pathogenesis and development of depression, new therapeutic tools have been experimentally tested in order to improve the current treatment of depressive disorders. In this context, hydroxylated and/or methoxylated aromatic acids have shown promising results as neuroprotective agents [25]. Among these acids, there are phenolic acids, which are divided into hydroxybenzoic acids and hydroxycinnamic acids, based on C_6_–C_1_ and C_6_–C_3_ skeletons, respectively [26]. According to the literature, phenolic acids have potentially antioxidant properties due to the presence of a phenolic ring that promotes the electron donation and hydrogen atom transfer to free radicals, acting as free-radical scavengers, reducing agents, and quenchers of single oxygen formation [27,28]. It has been reported that phenolic compounds produce a significant reduction of pro-inflammatory cytokines, including TNF-α and IL-1β, and stimulate a concomitant increase of anti-inflammatory cytokines as interleukin-8 (IL-8) in different in vitro and in vivo models of inflammation [29,30,31,32]. Here, the antidepressant effect of cinnamic acids was investigated, specifically the trimethoxycinnamic acids, *p*-coumaric acid, caffeic acid, and ferulic acid on animal models and their relevance to the treatment of depression. The last three compounds are classified as phenolic acids.

**Figure 3:**
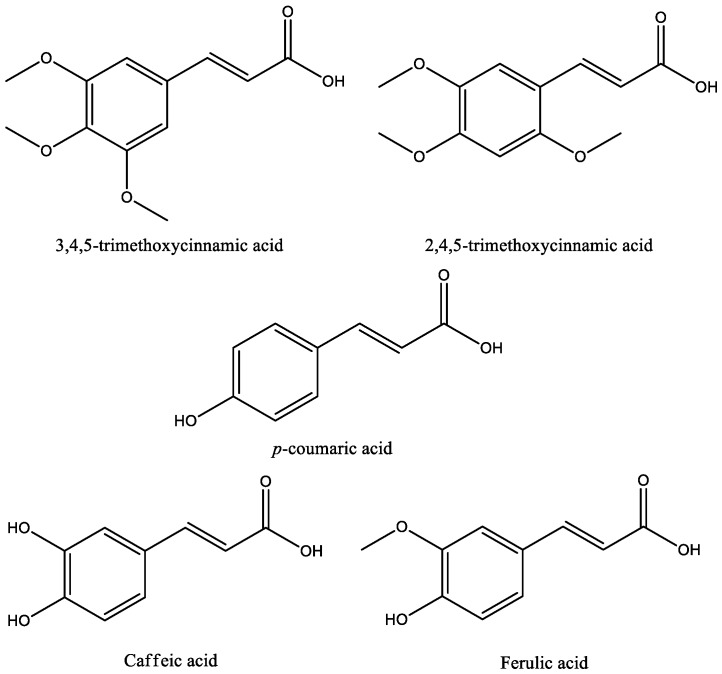
Chemical structures of antidepressant cinnamic acids.

Fifteen studies were found on cinnamic acids in experimental models of depression; the chemical structures of these acids are shown in Figure 3. Basically, the experimental model used in almost all studies for assessing antidepressant-like activity of cinnamic acids were the forced swim test (FST) and the tail suspension test (TST). Both tests are validated animal models fundamental for understanding the pathogenesis and treatment of mood and anxiety disorders, such as depression. The FST is based on the immersion of rodents in a beaker of water without a possible escape—a compound qualifies as a potential antidepressant if it reverts or delays the initial attempts to escape (active behavior) and promotes a progressive increase in the frequency and duration of episodes of immobile floating (passive behavior). The TST is based on the fact that animals subjected to the short-term, inescapable stress of being suspended by their tail, will develop an immobile posture [33,34]. Other important tests used to evaluate the effect of cinnamic acids on anxiety behaviors were the elevated plus maze (EPM) test, sucrose preference test, and open field test (OFT). In the EPM, the measure for anxiety is calculated by percentage of the total number of arm entries and the period of time spent on the open arms. Open-arm entries indicate security and low anxiety, while closed-arm entries, a sign of need for security. Decreased sucrose preference indicates the loss of the ability to feel pleasure (anhedonia), a common symptom of depression [34,35]. The OFT measures locomotor activity, including ambulation, exploration, latency, escape attempts, exploration, and the aversions of rodents to novel, brightly lit, open environments. In the OFT, reduced locomotor activity suggests anxiety-like behaviors associated with depression [34].

In the literature, two studies have reported the effects of trimethoxycinnamic acid (TMCA) on depressive behaviors, showing divergences in results. In Nakazawa et al. (2003), 2,4,5-trimethoxycinnamic acid failed to alter the duration of immobility in FST for Male ddY mice intraperitoneally treated with TMCA, at a dose ranging from 25–200 mg/kg. Differently, Leem and Oh (2015) have shown that Male C57BL/6J mice, orally treated with 3,4,5-trimethoxycinnamicacid (50 mg/kg) for 15 days, showed reduced immobility in the FST and higher time and frequency of visits in the open arms than the control group in the EPM. The data suggest an antidepressant effect of 3,4,5-trimethoxycinnamic acid, which the authors have attributed to increased expression of the ΔFosB protein on the nucleus accumbens observed in TMCA-treated animals. There were differences in some methodological aspects between the two investigative approaches. Thus, it is difficult to conclude about the presence or absence of antidepressant effect of TMCA based on only two experimental studies that used different routes of administration, species of mice, and experimental protocols. In addition, antidepressant activity may be related to the position of the methoxyl group in the aromatic ring, since animals treated with 3,4,5-trimethoxycinnamic acid had antidepressant effects, which did not occur in those treated with 2,4,5-trimethoxycinnamic acid [36,37].

Some studies performed by Takeda and his group have shown that male ICR mice subjected to intraperitoneal treatment with caffeic acid, at a dose of 4 mg/kg, exhibit decreased immobility time in the FST, as well as a reduction in the duration of freezing of mice in the conditioned fear stress test. According to authors, the caffeic acid’s ability to reduce depressive behavior might be attributed to, at least in part, an indirect modulation of the α1A-adrenoceptor and α1-adrenoceptor system and regulation of brain-derived neurotrophic factor (BDNF) expression in the frontal cortex caused by caffeic acid [38,39,40]. These data corroborate a study by Dzitoyeva and colleagues in which caffeic acid demonstrated antidepressant activity, attenuating BDNF mRNA decrease, by forced swimming test, using wild type mice and a 5-lipoxygenase (5-LOX) deficient group—previous studies have shown that caffeic acid inhibits 5-LOX. However, in this study it was observed that attenuation occurred only in wild rats, indicating that this acid can be used as a tool to study the regulation of the 5-LOX pathway of BDNF expression [41]. Therefore, antidepressant activity of caffeic acid may be related to its ability to regulate inflammation, as observed in the study by Huang et al. (2018), in which this acid inhibited dose-dependent increase in inflammatory and inducing cytokines affecting 5-HT, DA, and NE metabolism, such as tyrosine (Tyr), 3-methoxy-4-hydroxyphenylglycol (MHPG), Tryptophan (Trp), and 5-hydroxyindoleacetic acid (5-HIAA), improving the behavior of depressed rats [42].

The antidepressant activity of ferulic acid is the most investigated among hydroxycinnamic acids [32,43]. According to Zeni and colleagues, ferulic acid exhibits major effects on free radical and inflammatory messengers and has the ability to counteract the reduction in reward-seeking behavior that tends to occur with depression. Animals also exhibit decreased immobility time in the FST and TST, using ferulic acid oral treatment ranging from 0.001–80 mg/kg/day. Moreover, several studies show that ferulic acid has no effect on locomotor activity in the OFT, indicating its specificity, while others show a reversal of decreased locomotor activity, suggesting ferulic acid’s ability to reduce anxiety-like behaviors associated with depression. The antidepressant effect of ferulic acid has been attributed to diverse mechanisms, including modulation of serotonergic system by signaling pathway of protein kinase A (PKA), Ca^2+^/calmodulin-dependent protein kinase II (CaMKII), protein kinase C (PKC), mitogen-activated protein kinases/extracellular signal-regulated kinases (MAPK/ERK), and phosphoinositide 3-kinases (PI3K) [44,45].

Ferulic acid acts as some of the antidepressant drugs from the pharmaceutical market, as shown by the study by Chen et al. (2015), in which there is an increase on the concentrations of monoamines serotonin and norepinephrine in the hippocampus and frontal cortex through the inhibition of monoamine oxidase A (MAO-A) activity in male ICR mice treated with ferulic acid [46]. However, unlike commercially available selective serotonin reuptake inhibitor (SSRI) drugs, which may cause bowel movement inhibition, ferulic acid exhibited both antidepressant and prokinetic activity. The Zhang et al. study performed the TST on rats, noting a reduction in immobility time, and increased locomotor activity, associated with an increase in gastric emptying speed [47].

In addition, it was noted that oral administration of ferulic acid at a dose of 5 mg/kg in male ICR mice for 7 days decreased TST immobility due to up-regulation of gene expression associated with cell survival and proliferation, energy metabolism, and synthesis of dopamine in the limbic system of the brain of mice [48].

Lenziet al. (2015) have related ferulic acid’s antioxidant activity and its effects on the central nervous system, evidenced by increasing superoxide dismutase (SOD), catalase (CAT) activities, and low thiobarbituric acid reactive substances (TBARS) levels found in hippocampus of ferulic acid-treated male swiss mice [49]. Furthermore, Li et al. attributed the ferulic acid’s ability to reduce depressive-like behaviors, suggested by reducing immobility time in the FST and TST, observed in ferulic acid-treated male ICR mice to anti-inflammatory mechanisms [50]. In addition, a study of Liu et al. (2017) reported that ferulic acid increased the levels of BDNF and synaptic proteins (synapsin I and PSD-95) in the prefrontal cortex and hippocampus, as well as inhibited microglia activation, pro-inflammatory cytokines expression, nuclear factor kappa B (NF-κB) signaling, and decreased PYD domains-containing protein 3 NLRP3 [51].

A similar study by Zheng and colleagues demonstrated the relationship between the antidepressant effect and the anti-inflammatory activity of ferulic acid in prenatally-stressed offspring rats. In this work, it was noted that the administration of ferulic acid decreased the time of immobility and total number of crossing, rearing, grooming, and increased sucrose intake in the animals. Furthermore, it was able to reduce the concentration of inflammatory cytokines such as IL-6, IL-1, and TNF-α, and increase IL-10. As such, it was concluded that the antidepressant activity of ferulic acid occurs in part due to its anti-inflammatory activity and regulation on hypothalamic-pituitary-adrenal (HPA) axis [52].

In the study of Lee et al., it was observed that the antidepressant effects of *p*-coumaric acid may be related to its anti-inflammatory activity, as has also been shown in studies with caffeic acid. In this study, *p*-coumaric acid reduced lipopolysaccharide-induced tumor necrosis factor-α (LPS)-induced despair-related behavioral symptoms in the FST, TST, and sucrose splash test (SST), preventing the increase of inflammatory cytokines such as cyclooxygenase-2 and lipopolysaccharide-induced tumor necrosis factor-α (LPS), as well as inhibiting BDNF reduction [53].

Medicinal plants used to treat behavioral disorders that contain these psychoactive acids may have antidepressant action such as *Eugenia catharinensis* D. Legrand. Ethyl acetate extract of this species had antioxidant and antidepressant-like action in mice treated with corticosterone. Analysis of the extract using HPLC-ESI-MS/MS demonstrated the presence of several phenolic compounds, mainly phenolic acids, such as *p*-coumaric acid, ferulic acid, and caffeic acid. The authors suggest that chemical constituents are the active principles of antidepressant action [54]. Antidepressant action also was demonstrated for butanol fraction of *Olax subscorpioidea* Oliv. and was involved in the monoaminergic mechanism. Analysis of the chemical composition by HPLC indicated caffeic acid as one of the active ingredients of the plant [55]. In another study, ethyl acetate fraction from *Tabernaemontana catharinensis* A. DC. leaves showed antidepressant activity in animal models. Analysis of the chemical composition of this fraction proposes that the pharmacological action may be dependent on the presence of *p*-coumaric acid in the plant [56]. Therefore, the collection of studies discussed in this review (Table 1) show the pharmacological effect of cinnamic acids against the name Table 1 was added in text.

**Figure 4:**
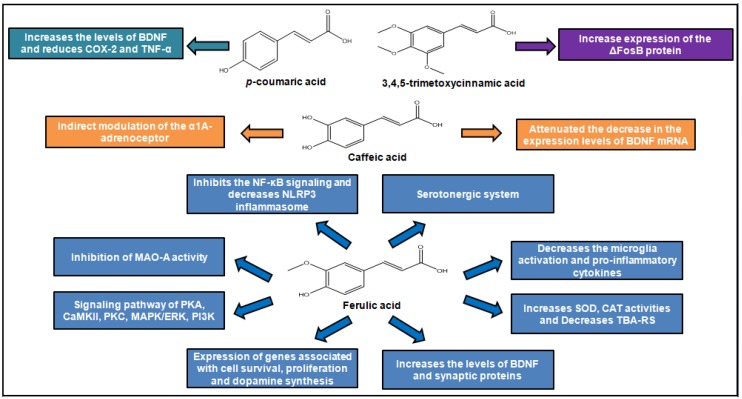
Antidepressant action of cinnamic acids.

Depressive disorders. Figure 4 summarizes the main effects of cinnamic acids as antidepressants.

## 4. Conclusions

Despite the few studies with cinnamic acids in animal depression models, the results indicate their potential applicability as candidates for antidepressant drugs. The studies discussed show that the antidepressant action of these natural products occurs via important neurotransmitters such as serotonin, as well as via the participation of inflammation-related metabolites such as AA-COX-2/5-LOX and BDNF. In some reports, there is a similarity in the mechanism of action with commercial antidepressant drugs. This data confirms the therapeutic potential of these compounds against behavioral disorders, such as depression. The availability of these compounds via commercial companies or laboratory synthesis, and the low cost of some acids, such as ferulic acid, make them interesting prototypes to advance the development of new antidepressant agents.

## Figures and Tables

**Table 1:** Cinnamic acids studied in experimental depression.

Compound	Animal Species	Dose and via of Administration	Behavioral Test	Observed Effects	Mechanism of Action	Reference
2,4,5-Trimethoxy- cinnamic acid (TMCA)	Male ddY mice	25, 50, 100 and 200 mg/kg, i.p.	FST	The treatment failed to alter the duration of immobility	-	[36]
3,4,5-Trimethoxy- cinnamic acid (TMCA)	Male C57BL/6J mice	25 and 50 mg/kg, p.o.	EPM; FST	The dose 50 mg/kg of treatment increased time and frequency of visits in the open arms of the EPM and showed reduced immobility in the FST	Increase expression of the ΔFosB protein on the nucleus accumbens	[37]
Caffeic acid	Male ICR mice	1, 2 and 4 mg/kg, i.p.	FST; spontaneous motor activity	The dose 4.0 mg/kg reduced the duration of immobility of mice	-	[38]
Caffeic acid	Male ICR mice and ddY mice	4 mg/kg,i.p.	Conditioned fear stress test	Reduced the duration of immobility of mice in the forced swimming test and reduced the duration of freezing of mice in the conditioned fear stress test	Indirect modulation of the α1A-adrenoceptor and α1-adrenoceptor system	[39]
Caffeic acid	Male ICR mice	4 mg/kg,i.p.	FST	Reduced the duration of immobility of mice in the forced swimming test	Attenuated the decrease in the expression levels of BDNF mRNA in the frontal cortex of mice following forced swimming	[40]
Caffeic acid	Male 5-LOX deficient mice and wild type	4 mg/kg,i.p.	FST	The pre-treatment was able to attenuate this decrease in the wild-type group	Caffeic acid can be used as a tool to study 5-lipoxygenase (5-LOX) pathway regulation of brain-derived neurotrophic factor (BDNF) expression	[41]
Caffeic acid	Male Sprague-Dawley rats	50, 75 and 100 mg/kg, i.p	OFT; FST	Inhibited the decrease of NE and the increase of Trp and MHPG in a dose-dependent manner	The inhibition of AA-COX-2/5-LOX pathways can improve the behaviors of depression rats	[42]
Ferulic acid	Male Sprague–Dawley rats	25 and 50 mg/kg, p.o.	FST; OFT	The dose 50 mg/kg reduced the duration of immobility of mice	Involvement of serotonergic system	[47]
Ferulic acid	Male Swiss mice	0.001, 0.01, 0.1, 1 and 10 mg/kg, p.o.	FST; TST; OFT	The doses 0.01, 0.1, 1 and 10 mg/kg reduced the duration of immobility of mice	Involvement of serotonergic system	[44]
Ferulic acid	Male Swiss mice	0.01 mg/kg, p.o.	TST; OFT	The dose 0.01 mg/kg reduced the duration of immobility of mice	Involvement signaling pathway of PKA, CaMKII, PKC, MAPK/ERK and PI3K	[44]
Ferulic acid	Male ICR mice	10, 20, 40 and 80 mg/kg, p.o.	FST; TST	The doses 40 and 80 mg/kg showed reduced immobility in the tests	The increase on the concentrations of monoamines 5-HT and norepinephrine in the hippocampus and frontal cortex through inhibition monoamine oxidase A (MAO-A) activity	[46]
Ferulic acid	Male Swiss mice	0.01, 0.1, 1 and 10 mg/kg/day, p.o.	FST; TST; OFT	The dose 1.0 mg/kg reduced the duration of immobility of mice	Increases SOD, CAT activities and decreases TBA-RS levels in hippocampus	[49]
Ferulic acid	Male ICR mice	3, 10, 30 and 90 mg/kg, p.o.	TST; FST; Locomotor activity	All doses reduced the duration of immobility of mice	-	[50]
Ferulic acid	Male ICR mice	20 and 40 mg/kg, p.o.	FST	The dose 40 mg/kg reduced the duration of immobility of mice	Increased the levels of BDNF and synaptic proteins (synapsin I and PSD-95) in both the prefrontal cortex and hippocampus.	[51]
Ferulic acid	Male ICR mice	20, 40 or 80 mg/kg, p.o.	SST; TST	All doses reduced the duration of immobility of mice	Inhibition of the microglia activation, pro-inflammatory cytokines expression, NF-κB signaling and decreased NLRP3 inflammasome	[51]
Ferulic acid	Male Swiss mice	1 mg/kg, p.o.	TST; OFT; SST	The dose 1.0 mg/kg reduced the duration of immobility of mice	-	[45]
Ferulic acid	Male ICR mice	5 mg/kg, p.o	TST	The dose 5 mg/kg reduced the duration of immobility of mice	Upregulates the expression of several genes associated with cell survival and proliferation, energy metabolism, and dopamine synthesis in mice limbic system of brain	[48]
Ferulic acid	Male Sprague-Dawley rats prenatally	12.5, 25, and 50 mg/kg, i.g.	SST, FST, OFT	Increased sucrose intake, and decreased immobility time and total number of crossings, rearing and grooming	Decreased concentration of inflammatory cytokines such as IL-6, IL-1 and TNF-α and increases IL-10	[52]
*p-*Coumaric acid	Male Sprague-Dawley rats	10 and 30 mg/kg p.o	FST; TST; SST	Improved LPS-induced despair-related behavioral symptoms	Prevented the increase of inflammatory cytokines in the hippocampus and the reduction of BDNF	[53]

## Abbreviations

5-HIAA	5-Hydroxyindoleacetic acid
5-HT	Serotonin
5-LOX	5-Lipoxygenase
BDNF	Brain-derived neurotrophic factor
CaMKII	Ca2^+^/calmodulin-dependent protein kinase II
CAT	Catalase
COX-2	Cyclooxygenase-2
EPM	Elevated plus maze test
FST	Forced swim test
IL-1β	Interleukin-1β
IL-8	Interleukin-8
LPS	Lipopolysaccharide
MAO	Monoamine oxidase
MAO-A	Monoamine oxidase A
MAPK/ERK	Mitogen-activated protein kinases/extracellular signal-regulated kinases
MHPG	3-Methoxy-4-hydroxyphenylglycol
NF-κB	Nuclear factor kappa B
NE	Norepinephrine
NET	Norepinephrine transporter
NO	Nitric oxide
OFT	Open field test
PI3K	Phosphoinositide 3-kinases
PKA	Protein kinase A
PKC	Protein kinase C
ROS	Reactive species of oxygen
SERT	Serotonin transporter
SNRI	Serotonin norepinephrine reuptake inhibitors
SOD	Superoxide dismutase
SSRI	Selective serotonin reuptake inhibitor
SST	Sucrose splash test
TBARS	Thiobarbituric acid reactive substances
TCA	Tricyclic antidepressants
TYR	Tyrosine
TMCA	2,4,5-Trimethoxycinnamic acid
TNF-α	Tumor necrosis factor-α
TRP	Tryptophan
TST	Tail suspension test

## References

[1] Peres M.F.P., Mercante J.P.P., Tobo P.R., Kamei H., Bigal M.E. (2017). Anxiety and depression symptoms and migraine: A symptom-based approach research. J. Headache Pain.

[2] Smith K. (2014). Mental health: A world of depression. Nature.

[3] Bravender T. (2018). Mental Disorders and Learning Disabilities in Children and Adolescents: Depression in Adolescents. FP Essent..

[4] Wang S., Blazer D.G. (2015). Depression and cognition in the elderly. Annu Rev. Clin. Psychol..

[5] Gournellis R., Tournikioti K., Touloumi G., Thomadakis C., Michalopoulou P.G., Michopoulos I., Christodoulou C., Papadopoulou A., Douzenis A. (2018). Psychotic (delusional) depression and completed suicide: A systematic review and meta-analysis. Ann. Gen. Psychiatry.

[6] Hawton K., Casanas I.C.C., Haw C., Saunders K. (2013). Risk factors for suicide in individuals with depression: A systematic review. J. Affect. Disord..

[7] Krishnan V., Nestler E.J. (2008). The molecular neurobiology of depression. Nature.

[8] Peng G.J., Tian J.S., Gao X.X., Zhou Y.Z., Qin X.M. (2015). Research on the Pathological Mechanism and Drug Treatment Mechanism of Depression. Curr. Neuropharmacol..

[9] Ruiz N.A.L., Del Angel D.S., Olguin H.J., Silva M.L. (2018). Neuroprogression: The hidden mechanism of depression. Neuropsychiatr. Dis. Treat..

[10] Uchida S., Yamagata H., Seki T., Watanabe Y. (2018). Epigenetic mechanisms of major depression: Targeting neuronal plasticity. Psychiatry Clin. Neurosci.

[11] Liu C.H., Zhang G.Z., Li B., Li M., Woelfer M., Walter M., Wang L. (2019). Role of inflammation in depression relapse. J. Neuroinflammation.

[12] Reus G.Z., Silva R.H., de Moura A.B., Presa J.F., Abelaira H.M., Abatti M., Vieira A., Pescador B., Michels M., Ignacio Z.M. (2019). Early Maternal Deprivation Induces Microglial Activation, Alters Glial Fibrillary Acidic Protein Immunoreactivity and Indoleamine 2,3-Dioxygenase during the Development of Offspring Rats. Mol. Neurobiol..

[13] Oglodek E.A. (2018). Changes in the concentrations of inflammatory and oxidative status biomediators (MIP-1 alpha, PMN elastase, MDA, and IL-12) in depressed patients with and without posttraumatic stress disorder. Pharmacol Rep..

[14] Stanners M.N., Barton C.A., Shakib S., Winefield H.R. (2014). Depression diagnosis and treatment amongst multimorbid patients: A thematic analysis. BMC Fam. Pract..

[15] Blackburn T.P. (2019). Depressive disorders: Treatment failures and poor prognosis over the last 50 years. Pharmacol. Res. Perspect..

[16] Hollon S.D., Cohen Z.D., Singla D.R., Andrews P.W. (2019). Recent Developments in the Treatment of Depression. Behav. Ther..

[17] López-Rubalcava C., Estrada-Camarena E. (2016). Mexican medicinal plants with anxiolytic or antidepressant activity: Focus on preclinical research. J. Ethnopharmacol..

[18] Guzman J.D., Mortazavi P.N., Munshi T., Evangelopoulos D., Mchugh T.D., Gibbons S., Malkinson J., Bhakta S. (2014). 2-Hydroxy-substituted cinnamic acids and acetanilides are selective growth inhibitors of Mycobacterium tuberculosis. MedChemComm.

[19] Tian Y., Liu W., Lu Y., Wang Y., Chen X., Bai S., Zhao Y., He T., Lao F., Shang Y. (2016). Naturally Occurring Cinnamic Acid Sugar Ester Derivatives. Molecules.

[20] de Cássia D.S.E.S., Andrade L.N., Dos Reis B.D.O.R., de Sousa D.P. (2014). A review on anti-inflammatory activity of phenylpropanoids found in essential oils. Molecules.

[21] Sova M. (2012). Antioxidant and antimicrobial activities of cinnamic acid derivatives. Mini Rev. Med. Chem..

[22] Anantharaju P.G., Gowda P.C., Vimalambike M.G., Madhunapantula S.V. (2016). An overview on the role of dietary phenolics for the treatment of cancers. Nutr. J..

[23] Alam M.A., Subhan N., Hossain H., Hossain M., Reza H.M., Rahman M.M., Ullah M.O. (2016). Hydroxycinnamic acid derivatives: A potential class of natural compounds for the management of lipid metabolism and obesity. Nutr. Metab..

[24] Liu P., Hu Y., Guo D.H., Wang D.X., Tu H.H., Ma L., Xie T.T., Kong L.Y. (2010). Potential antidepressant properties of Radix polygalae (Yuan Zhi). Phytomedicine.

[25] Szwajgier D., Borowiec K., Pustelniak K. (2017). The Neuroprotective Effects of Phenolic Acids: Molecular Mechanism of Action. Nutrients.

[26] Magoulas G.E., Papaioannou D. (2014). Bioinspired syntheses of dimeric hydroxycinnamic acids (lignans) and hybrids, using phenol oxidative coupling as key reaction, and medicinal significance thereof. Molecules.

[27] Bialecka-Florjanczyk E., Fabiszewska A., Zieniuk B. (2018). Phenolic Acids Derivatives-Biotechnological Methods of Synthesis and Bioactivity. Curr. Pharm. Biotechnol..

[28] Wu S., Zhang Y., Ren F., Qin Y., Liu J., Liu J., Wang Q., Zhang H. (2018). Structure-affinity relationship of the interaction between phenolic acids and their derivatives and beta-lactoglobulin and effect on antioxidant activity. Food Chem..

[29] Dludla P.V., Nkambule B.B., Jack B., Mkandla Z., Mutize T., Silvestri S., Orlando P., Tiano L., Louw J., Mazibuko-Mbeje S.E. (2019). Inflammation and Oxidative Stress in an Obese State and the Protective Effects of Gallic Acid. Nutrients.

[30] Oliviero F., Scanu A., Zamudio-Cuevas Y., Punzi L., Spinella P. (2018). Anti-inflammatory effects of polyphenols in arthritis. J. Sci. Food Agric..

[31] Gaspar A., Garrido E.M., Esteves M., Quezada E., Milhazes N., Garrido J., Borges F. (2009). New insights into the antioxidant activity of hydroxycinnamic acids: Synthesis and physicochemical characterization of novel halogenated derivatives. Eur. J. Med. Chem..

[32] Razzaghi-Asl N., Garrido J., Khazraei H., Borges F., Firuzi O. (2013). Antioxidant properties of hydroxycinnamic acids: A review of structure- activity relationships. Curr. Med. Chem..

[33] Castagne V., Moser P., Roux S., Porsolt R.D. (2011). Rodent models of depression: Forced swim and tail suspension behavioral despair tests in rats and mice. Curr. Protoc. Neurosci..

[34] Yan H.C., Cao X., Das M., Zhu X.H., Gao T.M. (2010). Behavioral animal models of depression. Neurosci. Bull..

[35] Abelaira H.M., Reus G.Z., Quevedo J. (2013). Animal models as tools to study the pathophysiology of depression. Braz. J. Psychiatry.

[36] Nakazawa T., Yasuda T., Ueda J., Ohsawa K. (2003). Antidepressant-like effects of apigenin and 2, 4, 5-trimethoxycinnamic acid from Perilla frutescens in the forced swimming test. Biol. Pharm. Bull..

[37] Leem Y.H., Oh S. (2015). 3, 4, 5-Trimethoxycinnamin acid ameliorates restraint stress-induced anxiety and depression. Neurosci. Lett..

[38] Takeda H., Tsuji M., Inazu M., Egashira T., Matsumiya T. (2002). Rosmarinic acid and caffeic acid produce antidepressive-like effect in the forced swimming test in mice. Eur. J. Pharmacol..

[39] Takeda H., Tsuji M., Miyamoto J., Masuya J., Iimori M., Matsumiya T. (2003). Caffeic acid produces antidepressive-and/or anxiolytic-like effects through indirect modulation of the α1A-adrenoceptor system in mice. Neuroreport.

[40] Takeda H., Tsuji M., Yamada T., Masuya J., Matsushita K., Tahara M., Matsumiya T., Limore M., Matsumiya T. (2006). Caffeic acid attenuates the decrease in cortical BDNF mRNA expression induced by exposure to forced swimming stress in mice. Eur. J. Pharmacol..

[41] Dzitoyeva S., Imbesi M., Uz T., Dimitrijevic N., Manev H., Manev R. (2008). Caffeic acid attenuates the decrease of cortical BDNF transcript IV mRNA induced by swim stress in wild-type but not in 5-lipoxygenase-deficient mice. J. Neural Transm. Suppl..

[42] Huang D., Zhang L., Yang J.Q., Luo Y., Cui T., Du T.T., Jiang X.H. (2019). Evaluation on monoamine neurotransmitters changes in depression rats given with sertraline, meloxicam or/and caffeic acid. Genes Dis..

[43] Zdunska K., Dana A., Kolodziejczak A., Rotsztejn H. (2018). Antioxidant Properties of Ferulic Acid and Its Possible Application. Skin Pharmacol. Physiol..

[44] Zeni A.L.B., Zomkowski A.D.E., Maraschin M., Rodrigues A.L.S., Tasca C.I. (2012). Ferulic acid exerts antidepressant-like effect in the tail suspension test in mice: Evidence for the involvement of the serotonergic system. Eur. J. Pharmacol..

[45] Zeni A.L.B., Camargo A., Dalmagro A.P. (2017). Ferulic acid reverses depression-like behavior and oxidative stress induced by chronic corticosterone treatment in mice. Steroids.

[46] Chen J., Lin D., Zhang C., Li G., Zhang N., Ruan L., Yan K., Li J., Yu X., Xie X. (2015). Antidepressant-like effects of ferulic acid: Involvement of serotonergic and norepinergic systems. Metab. Brain Dis..

[47] Zhang Y.J., Huang X., Wang Y., Xie Y., Qiu X.J., Ren P., Gao L.C., Zhou H.H., Zhange H.Y., Qiao M.Q. (2011). Ferulic acid-induced anti-depression and prokinetics similar to Chaihu–Shugan–San via polypharmacology. Brain Res. Bull..

[48] Sasaki K., Iwata N., Ferdousi F., Isoda H. (2019). Antidepressant-Like Effect of Ferulic Acid via Promotion of Energy Metabolism Activity. Mol. Nutr. Food Res..

[49] Lenzi J., Rodrigues A.F., de Sousa Rós A., de Castro B.B., de Lima D.D., Dal Magro D.D., Zeni A.L.B. (2015). Ferulic acid chronic treatment exerts antidepressant-like effect: Role of antioxidant defense system. Metab. Brain Dis..

[50] Li G., Ruan L., Chen R., Wang R., Xie X., Zhang M., Chen L., Yan Q., Reed M., Chen J. (2015). Synergistic antidepressant-like effect of ferulic acid in combination with piperine: Involvement of monoaminergic system. Metab. Brain Dis..

[51] Liu Y.M., Hu C.Y., Shen J.D., Wu S.H., Li Y.C., Yi L.T. (2017). Elevation of synaptic protein is associated with the antidepressant-like effects of ferulic acid in a chronic model of depression. Physiol. Behav..

[52] Zheng X., Cheng Y., Chen Y., Yue Y., Li Y., Xia S., Li Y., Deng H., Zang J., Cao Y. (2019). Ferulic Acid Improves Depressive-Like Behavior in Prenatally-Stressed Offspring Rats via Anti-Inflammatory Activity and HPA Axis. Int. J. Mol. Sci..

[53] Lee S., Kim H.B., Hwang E.S., Kim E.S., Kim S.S., Jeon T.D., Song M., Li J.S., Chung M.C., Maeng S. (2018). Antidepressant-like effects of *p*-coumaric acid on LPS-induced depressive and inflammatory changes in rats. Exp. Neurobiol..

[54] Barauna S.C., Delwing-Dal M.D., Brueckheimer M.B., Maia T.P., Sala G.A.B.N., Döhler A.W., Harger M.C., de Melo D.F.M., de Gasper A.L., Alberton M.D. (2018). Antioxidant and antidepressant-like effects of *Eugenia catharinensis* D. Legrand in an animal model of depression induced by corticosterone. Metab. Brain Dis..

[55] Adeoluwa A.O., Aderibigbe O.A., Agboola I.O., Olonode T.E., Ben-Azu B. (2019). Butanol Fraction of *Olax subscorpioidea* Produces Antidepressant Effect: Evidence for the Involvement of Monoaminergic Neurotransmission. Drug Res. (Stuttg)..

[56] Pauleti N.N., Mello J., Siebert D.A., Micke G.A., de Albuquerque C.A.C., Alberton M., Barauna S.C. (2018). Characterisation of phenolic compounds of the ethyl acetate fraction from *Tabernaemontana catharinensis* and its potential antidepressant-like effect. Nat. Prod. Res..

